# Soy Consumption and the Risk of Developing Bladder Cancer: A Pooled Analysis of Cohort Studies from the International Pooled Bladder Cancer Epidemiology and Nutritional Determinants (BLEND) Study

**DOI:** 10.3390/nu18193110

**Published:** 2026-09-22

**Authors:** Yue-Wei Wu, Evan Yi-Wen Yu, Ya-Ting Chen, Meng-Han Li, Yu-Ting Liang

**Affiliations:** 1 Key Laboratory of Environmental Medicine and Engineering of the Ministry of Education, School of Public Health, Southeast University, Nanjing 210009, China; 2 Department of Epidemiology & Biostatistics, School of Public Health, Southeast University, Nanjing 210009, China; 3 Department of Epidemiology, CAPHRI Care and Public Health Research Institute, Maastricht University, 6229 ER Maastricht, The Netherlands

**Keywords:** soy, bladder cancer, risk, prospective analysis

## Abstract

**Background:** Epidemiological studies have shown inconsistent results in associations between soy consumption and bladder cancer (BC). Therefore, this research aims to find more conclusive answers on the influence of soy consumption on the development of BC risk by bringing together 12 worldwide available cohort studies on this topic. **Materials and Methods:** The risk of developing BC was investigated in a pooled analysis of 12 cohort studies comprising 549,458 participants, including 3164 incident BC cases. Cox proportional hazard regression analysis was used to obtain the pooled hazard ratios (HRs) and corresponding 95% confidence intervals (CIs). **Results:** No association between total soy intake and risk of development of BC was observed. However, after adjustment for potential confounders, soybean oil intake was associated with an increased risk (HR_model2_ = 1.49, 95% CI 1.03–2.16) comparing never consumption with ever consumption as well as with moderate (HR_model2_ 1.69, 95% CI 1.08–2.67; HR_model3_ 1.65, 95% CI 1.03–2.64) and high (HR_model2_ 1.57, 95% CI 1.06–2.33; HR_model3_ 1.50, 95% CI 1.00–2.26) consumption. Overall, no significant associations were observed for soybean, soy milk or unfermented soy paste intake. For females a significant decreased BC risk for moderate soybean consumption was observed (HR = 0.46, 95% CI 0.22–0.89). For males a significant increased BC risk in ever soybean oil consumers as well as in high soy consumers was found (HR = 1.63, 95% CI 1.00–2.66; HR = 1.69, 95% CI 1.02–2.80, respectively). **Conclusions:** This research suggests that different sub-categories of soy consumption may have different associations with BC risk, showing differences between sexes exist.

## 1. Introduction

Bladder cancer (BC) is the tenth leading cancer worldwide, with an estimated 549,393 new cases and 199,922 deaths in 2018. In addition, bladder cancer is the thirteenth most deadly cancer worldwide [1]. Seventy five percent of all BC cases occur in male [1,2,3,4] and Europe is known to have the highest incidence rates for BC [2]. Due to the fact that this type of cancer is easy to relapse, BC is reported to be the most expensive life-time treatment of all cancers [5,6,7,8].

The main risk factors for BC are tobacco smoking [2,3,9,10,11,12,13,14,15], exposure to high levels of arsenic in the drinking water [2,3,11] and infection caused by Schistosoma Haematobium [2,16]. Since the bladder is an excretion organ, dietary factors might also influence the BC risk. Both healthful and harmful components of the diet are excreted through the urinary tract and come into contact with the epithelium of the bladder [3,17,18], thereby potentially affecting the development of BC [19]. Although several food items have already been shown to affect the BC risk [10,12,17,20,21,22,23], the WCRF/AICR (World Cancer Research Fund/American Institute for Cancer Research) stated that there is still limited evidence for the role of diet in BC development [24].

An interesting nutritional component in terms of cancer development is soy. Soy-derived isoflavones, particularly genistein and daidzein, may influence carcinogenesis through several biological pathways [25,26], which are known as antioxidants with antitumorigenic effects [4,15,25,26,27]. Because of their structural similarity to endogenous oestrogens, these compounds can interact with both oestrogen receptor α (ERα) and oestrogen receptor β (ERβ), with preferential activation of ERβ [28]. Oestrogen-receptor signalling has been implicated in urothelial carcinogenesis and tumour progression, although ERα and ERβ may have different biological effects [29]. In addition, the gut microbiota can metabolise daidzein to equol, contributing to interindividual differences in the bioavailability and biological activity of soy-derived phytoestrogens [30]. Through these and other pathways, soy isoflavones may modulate cancer metastasis inhibition properties [18,31,32,33,34] and have a shown effect on cell cycle progression of BC cells (i.e., enhance apoptosis and inhibit cell proliferation) [18,26,31,34]. However, although this potential beneficial effect of soy on BC development, in our knowledge only two epidemiological studies investigated the effect of a diet high in soy foods on BC risk, with inconsistent results. While the Takayama cohort study [4] found a significant inverse association between BC risk and high intake of total soy and isoflavones among males, a study conducted in Singapore found an increased BC risk associated with high intake of total soy and isoflavones [35].

Previous prospective studies examining the association between soy consumption and bladder cancer risk remain limited, and their findings have been inconsistent. In addition, most epidemiological evidence on soy consumption and bladder cancer has come from Asian populations, where habitual soy intake is relatively high. Evidence from Western populations, where soy consumption is generally lower, remains limited. Therefore, large-scale pooled analyses using individual-level data are needed to clarify the associations of total soy intake and different soy-based foods with bladder cancer risk.

To increase our understanding and to find more conclusive answers on the influence of soy consumption on the development of BC risk, the present study aimed to investigate the association between soy and BC risk by aggregating available cohort studies on this topic, including 3164 BC cases.

## 2. Materials and Methods

### 2.1. Study Population

Data were used from the Bladder Cancer Epidemiology and Nutritional Determinants study (BLEND). BLEND is a large international nutritional epidemiology consortium, which consists of cohort and case–control studies including populations from Europe, Asia and USA. BLEND aimed to pool and evaluate data available from epidemiological studies on individual food products, dietary patterns, nutrients and the risk of BC. For the present study, 12 cohort studies (including 3164 cases and 546,294 controls) had sufficient information on soy consumption to be eligible for inclusion in the analyses. Among participants with available ethnicity data, 545,443 (99.3%) were Caucasian. Overall soy consumption was relatively low, with a mean intake of 3 g/day; 69.3% of participants reported no soy consumption, whereas only 17.8% reported moderate or high consumption.

### 2.2. Data Collection and Coding

The BLEND consortium is an international pooled study investigating dietary factors and bladder cancer [36]. For the present analysis, 12 prospective cohorts with sufficient information on soy and soy-containing food consumption were eligible for inclusion. Briefly, the individual-level data from the included studies were combined into one dataset. To improve comparability, soy consumption was recoded using the Eurocode 2 food coding system developed by the European Union [37] and mapped to standardised categories of total soy and individual soy foods. Dietary intake was assessed using cohort-specific self-administered or interviewer-administered food frequency questionnaires (FFQs), and the reported weekly, monthly or yearly portion-based intake of soy and other dietary items was checked and converted to grams per day using standard portion sizes obtained from USDA FoodData Central [38]. In addition to information on dietary intake, the BLEND dataset also included data on study characteristics (design, method of dietary assessment, recall time of dietary intake and geographical region), participant demographics (age, sex, and ethnic group), TNM classification of Malignant Tumours (tumour node metastasis; TNM), and smoking status (current/former/never), smoking frequency and smoking duration at baseline [36].

### 2.3. Statistical Analyses

Differences in baseline between cases and non-cases, the different sub-categories of soy consumption and potential confounders were examined using the chi-square test for categorical variables and *t* test for continuous variables. Cox proportional hazard regression analyses were used to estimate the pooled hazard ratios (HRs) and 95% confidence intervals (CIs) to assess the influence of soy consumption on BC risk with time of recruitment as starting point. Person-year follow-up of the participants was calculated from the date that they entered to the date that participants were diagnosed with BC or the date when follow-up was ended due to other circumstances (such as death, lost to follow-up or study exit).

Soy consumption was indicated as follows: (1) ever soy consumption (individuals consumed soy > 0 g per day [g/day] or never soy consumption; (2) based on quartile ordered distribution, for total soy consumption this was divided in a never consumption group and the following three ever consumption groups: light soy consumption (>0–<1 g/day), moderate soy consumption (≥1–<10 g/day) and high soy consumption (≥10 g/day). For the different sub-categories of soy consumption, this was divided in the lowest tertile, middle tertile and highest tertile. Enough data were available for the following sub-categories and were assessed on their association with BC risk: soybean, soybean oil, soy milk and unfermented soy paste. The Cox regression models used never consumption as the reference group and were computed as crude model (model 1), adjusted for sex, age, smoking status (current, former, and never smoker), smoking pack-years and total energy intake (kcal/day) (model 2), or fully adjustment, additionally adjusted with vegetable intake (g/day) and fruit intake (g/day) (model 3). Smoking was described as 0 (never smokers), 1 (current smokers less than 20 pack-years), 2 (current smokers more than 20 pack-years), 3 (former smokers less than 20 pack-years), 4 (former smokers more than 20 packs-years), 5 (current smokers), and 6 (former smokers). To look into effect modification, interaction terms between soy consumption sex and smoking status were added to the models. *p* interaction < 0.05 was considered statistically significant, where upon all analyses were stratified for the covariate of interest.

In the secondary analysis, a possible dose-dependent relationship between soy consumption and BC was assessed by using fractional polynomial regression. Dose–response analyses results were presented for each decile with daily increment form the lowest decile up to the tenth decile in g/day. No imputation was performed for missing data. For each regression model, a complete-case approach was applied, whereby participants with missing information on the exposure, outcome or covariates included in that model were excluded from the corresponding analysis.

All statistical analyses were performed with STATA version 14.2 SE. *p* values < 0.05 were considered statistically significant.

## 3. Results

### 3.1. Baseline Characteristics

Baseline characteristics of the study population are shown in Table 1 and Table 2. In total, 3164 incident cases of BC (2376 males, 788 females) and 546,294 non-cases with a total follow up of 6,095,268 person-years were included in the analysis. Mean age at baseline was 60.5 (7.3) years for BC cases and 53 (10) years for non-cases. The majority of participants were Caucasian (545,443 participants) and only 3735 (0.7%) non-Caucasian (i.e., Asian, Black, Indian, and any other ethnic group). Approximately 18% of the study population reported soy consumption of moderate or high proportions, with an average consumption of 3 g/day. Of the cases, an average soy consumption of 3 g/day is reported, for non-cases the daily intake is 3 g. At baseline, a higher soy consumption was observed among male (4 g/day vs. 3 g/day for female) and among never and former smokers (respectively 4 g/day and 3 g/day vs. 2 g/day for current smokers). Non-Caucasians reported a significantly higher soy consumption (14 g/day) than Caucasian participants (3 g/day). No interaction was found between sex and smoking (*p* interaction: 0.53, 0.80, respectively).

### 3.2. Association Between Soy Consumption and BC Risk

#### 3.2.1. Ever Versus Never Soy Consumption

The results comparing ever versus never soy consumers are shown in Table 3. For total soy consumption, no statistically significant association with BC risk was found in models 2 and 3 (HR_model 2_ 1.00, 95% CI 0.87–1.15; HR_model 3_ 1.00, 95% CI 0.86–1.14). Although a slightly protective effect on BC risk was observed for soybean consumption, none of the associations showed to be statistically significant. A similar non-association was observed for soy milk. Soybean oil intake was associated with an increased BC risk in model 2 (HR_model 2_ 1.49, 95% CI 1.03–2.16). However, the association became marginally non-significant in model 3 (HR_model 3_ 1.46, 95% CI 0.99–2.14).

#### 3.2.2. Categories of Soy Consumptions

The results of the Cox proportional hazard regression analyses for sub-categories of soy consumption are shown in Table 3. For soybean oil consumption, a statistically significant increased risk of BC was observed for moderate (HR_model2_ 1.69, 95% CI 1.08–2.67; HR_model3_ 1.65, 95% CI 1.03–2.64) and high (HR_model2_ 1.57, 95% CI 1.06–2.33; HR_model3_ 1.50, 95% CI 1.00–2.26) consumption. High soybean intake showed a slightly protective effect on BC risk; however, this association was not statistically significant. Similar non-significant results were observed for soy milk intake. Unfermented soy paste intake appears to increase the BC risk, but none of the associations were statistically significant.

#### 3.2.3. Soy Consumption and BC Risk Stratified by Sex

The results for the sub-categories of soy consumption stratified by sex are shown in Table 4. For males, a significant increased BC risk was observed in ever soybean oil consumers compared to never soybean oil consumers (HR_model2_ 1.63, 95% CI 1.00–2.66), while no association was found among females. In addition, similar results were found comparing high soybean oil consumption with no soybean oil consumption in males (HR_model2_ 1.69, 95% CI 1.02–2.80). For females, a statistically significant decreased risk of BC was observed for moderate soybean consumption (HR_model2_ 0.46, 95% CI 0.22–0.89), while no such significant decrease in BC risk could be observed in males.

## 4. Discussion

This pooled analysis, including 12 cohort studies from various continents and countries, found no statistically significant association between total soy intake and risk of development of BC. Furthermore, an overall increased BC risk was observed for soybean oil intake.

Results of previous epidemiological studies on the association of soy consumption and BC risk were inconsistent. While the Takayama study including 31,552 participants [4] found a significant decreased BC risk with the highest soy intake among males, the Singapore health study based on approximately 63,000 participants showed a strong positive association between high total soy intake and BC risk [39]. The present study, however, found no association between total soy intake and BC risk. Since results from our sub-categories show differences in their relation with BC risk (i.e., soybean oil significantly increases the risk for BC, while soybean intake and soy milk intake slightly decrease the BC risk), these contrary finding might be due to differences in the soy sources asked in the questionnaires. The differences between our findings and those of previous Asian studies should also be interpreted in light of the exposure distribution and geographical composition of the present study population [4,35,39]. Most participants were drawn from Western populations, where soy and isoflavone consumption is generally lower than that reported in many Asian populations [40,41]. Although soy intake was generally lower in the predominantly Western study population, this exposure distribution provides complementary evidence on the association between soy consumption and bladder cancer at lower habitual intake levels. Nevertheless, the relatively limited exposure range may have reduced the ability to detect modest associations or effects that emerge only at higher levels of soy consumption. In addition, the types of soy foods consumed and their isoflavone contents may differ substantially across populations and according to food processing and preparation methods [42]. Therefore, the observed null association should not be interpreted as evidence of an absence of association in populations with substantially higher soy intake. Further prospective studies covering a wider range of soy consumption, particularly in Asian populations, are needed to assess the generalisability of these findings.

Soybean oil contains high levels of linoleic acid as their major fatty acid, particularly during high-temperature cooking or repeated heating, potentially resulting in the formation of lipid-oxidation products [43,44]. However, soybean oil contains negligible amounts of isoflavones; therefore, the observed positive association is unlikely to be explained by the isoflavone-related mechanisms proposed for other soy foods. Linoleic acid has been linked to development of cancer in animals [43] and may be prone to oxidation, which may play a role in carcinogenesis [43,45]. Moreover, soybean oil intake may serve as a marker of broader dietary patterns, including a higher consumption of fried, processed or energy-dense foods, rather than representing an independent effect of soybean oil itself. It may also be associated with lifestyle or socioeconomic characteristics that were not fully captured by the available covariates. Residual confounding, exposure measurement error and chance arising from multiple subgroup comparisons may therefore provide alternative explanations for the observed association. Accordingly, the positive association between soybean oil intake and BC risk should be interpreted cautiously and should not be considered evidence of a causal effect. Further studies with more detailed information on cooking methods, overall dietary patterns and relevant lifestyle factors are needed to confirm this finding. Soybeans and soy milk contain several biologically active constituents, among which the isoflavones genistein and daidzein have been proposed as important contributors to their potential anticancer properties [4,12,18,31,46]. Experimental studies have shown that genistein exerts antiproliferative effects on cancer cells and may protect cells against reactive oxygen species by scavenging free radicals and reducing the expression of stress-response-related genes [4,18,26,27,32,47]. The potential preventive effect of soybean-derived isoflavones on BC risk is further supported by experimental evidence showing that genistein may promote apoptosis and induce dose-dependent G2/M-phase cell-cycle arrest [18,27,32]. Soy isoflavones and their metabolites are also excreted in human urine and may therefore come into direct contact with the urothelium lining the bladder [17,26,31,46]. This direct exposure may allow soy-derived isoflavones to modulate cell proliferation, apoptosis, oxidative stress and angiogenesis in the urothelium, thereby potentially inhibiting the initiation or progression of bladder carcinogenesis [17,18,25,27]. However, in the present study no associations between intakes of soybeans and soy milk and BC were observed. This apparent inconsistency may partly reflect differences in the dose, bioavailability and metabolism of isoflavones, as well as the use of soybean and soy milk intake as indirect proxies for exposure to specific bioactive compounds. Because genistein, daidzein and their circulating or urinary metabolites were not directly measured, these biological mechanisms could not be evaluated in the present study.

Fermented soy products contain high amounts of isoflavones. During the fermentation process of isoflavones high levels of salt and *N*-nitroso compounds form, which are known to be excreted via the bladder and have carcinogenic effects [25,48,49]. Therefore, after fermentation the beneficial effects of soy can be masked [50,51]. In addition, fermented soy isoflavone aglycones have a faster absorption rate compared to isoflavones in unfermented soy isoflavones [52], which might also explain the differences in the observed finings between the different findings for un- and fermented soy products. Since no previous epidemiological studies investigated the difference between fermented and unfermented soy intake in relation to BC, the true association remains unclear and should be further investigated.

In the present study, sex stratification results showed different findings for males and females. In males, a significant increased BC risk was observed for soybean oil consumers, while among females a significant decreased BC risk was found for moderate soybean consumption. One possible explanation is that there might be a difference between sexes in exposure to potential carcinogens [53]. No uniform theory about the exposure differences between males and females exist, but environmental exposure to carcinogens such as tobacco and industrial chemicals has been suggested to be higher among male [54]. In addition, genetic, anatomical, hormonal and social factors are expected to play a role in the differences between males and females [54].

For the present study, data were collected from 12 cohort studies, originating from 10 different countries on three different continents, thereby obtaining a large sample size, provides statistical power to perform detailed and accurate analyses. However, our study has several limitations. First, limited information on possible confounding BC risk factors, other than age, sex, smoking and energy intake were available, such as Body Mass Index (BMI), physical activity, socioeconomic status, arsenic drinking water, Schistosoma Haematobium infection, level of education and occupational exposures to potentially carcinogenic chemicals. Additional adjustment for these potential confounding factors could have affected the results of the present study, although the previous literature suggested only proportion of BC cases can be attributed to these factors [53,55]. Another limitation of our study includes the conversion of the provided data on soy consumption from weekly, monthly or yearly intakes by portion to daily intake by grams. The conversion was done using the USDA FoodData Central, making the conversions not-country- or -cohort-specific. Size of standard soy portions is varied around the world. Therefore, the effect of a standard portion of soy on BC risk might differ between different countries. In addition, potential misclassification or measurement errors of portion size, including over- and underreporting of soy consumption, and frequency of food consumption derived from food frequency questionnaires (FFQs) could lead to errors estimating soy consumption within individual studies. Although the dietary data were harmonised using a common food-classification system and standardised intake definitions, residual between-cohort differences and exposure misclassification could not be completely eliminated. Residual confounding, differences in broader dietary and lifestyle patterns, exposure measurement error, and false-positive findings arising from multiple comparisons cannot be excluded. In addition, information of soy consumption was collected at baseline, but possible changes in dietary and lifestyle habits during the follow-up period are unavoidable. An additional limitation is that the intake of individual soy isoflavones, particularly genistein and daidzein, was not directly assessed. Soybean and soy milk intake may be imprecise proxies for isoflavone exposure because the concentrations of these compounds differ among soy products and according to processing and preparation methods [52]. The relatively small number of cancer cases in certain sex-specific subgroups may have limited statistical power and reduced the precision of the corresponding estimates, as reflected by the wider confidence intervals. Therefore, these subgroup-specific findings should be interpreted cautiously and require confirmation in larger studies with sufficient numbers of cases. Another limitation is that dietary intake was primarily assessed at baseline. Dietary habits may change during long-term follow-up, and a single baseline assessment may therefore become less representative of habitual intake over time. Such within-person changes in exposure may lead to regression dilution and attenuate associations toward the null, particularly in cohorts with extended follow-up [56]. Consequently, modest associations between soy consumption and bladder cancer risk may have been underestimated. Furthermore, dietary intake may not accurately reflect biologically available exposure because of interindividual differences in the absorption and metabolism of isoflavones. The lack of information on individual isoflavones and their circulating or urinary metabolites prevented us from determining whether the observed associations were attributable to these specific bioactive compounds.

## 5. Conclusions

In summary, no statistically significant association was observed between total soy intake and BC risk. Although a positive association was observed for soybean oil intake and sex-specific associations were observed for soybean and soybean oil intake, these findings should be interpreted cautiously. Therefore, the findings for individual soy products should be regarded as exploratory rather than evidence of causal effects. Further prospective studies involving populations with higher and more diverse soy consumption are needed, with detailed assessments of individual isoflavones, particularly genistein and daidzein, and adjustment for a broader range of potential confounding factors.

## Figures and Tables

**Table 1:** Baseline characteristics of the study population (3164 cases and 546,294 non-cases) and soy consumption category according to age, sex and smoking status.

Characteristics	Total	No Soy Consumption (0 g/Day)	Light Soy Consumption (>0–<1 g/Day)	Moderate Soy Consumption (≥1–<10 g/Day)	High Soy Consumption (≥10–<1600 g/Day)	Mean (g/Day)	SD	*p* Value	*p* Interaction
N (%)	549,458	380,771 (69.3)	70,903 (12.9)	65,975 (12.0)	31,809 (5.8)	3	23		
Case (%)	3164	2649 (83.7)	151 (4.8)	219 (6.9)	145 (4.6)	3	22		
Non-case (%)	546,294	378,122 (69.2)	70,752 (13.0)	65,756 (12.0)	31,664 (5.8)	3	23	<0.001 ***	
Person-years (SE)	6,095,268 (2900)	4,295,896 (2600)	765,597.9 (620)	702,318.7 (690)	331,454.6 (850)				
Sex									0.53
Male (%)	178,424	130,871 (73.3)	13,126 (7.4)	20,956 (11.7)	13,471 (7.5)	4	23		
Case	2376	2055 (86.5)	70 (2.9)	140 (5.9)	111 (4.7)	2	18		
Non-case	176,048	128,816 (73.2)	13,056 (7.4)	20,816 (11.8)	13,360 (7.6)	4	23	<0.001 ***	
Female (%)	371,034	249,900 (67.4)	57,777 (15.6)	45,019 (12.1)	18,338 (4.9)	3	23		
Case	788	594 (75.4)	81 (10.3)	79 (10.0)	34 (4.3)	4	30		
Non-case	370,246	249,306 (67.3)	57,696 (15.6)	44,940 (12.1)	18,304 (4.9)	3	23	<0.001 ***	
Age [mean (SD)]									0.62
Case	60.5 (7.3)	60.9 (6.9)	59 (11)	57.4 (8.7)	58.2 (7.3)	60.45	7.32		
Non-case	53 (10)	54.3 (8.8)	48 (11)	47 (12)	51.9 (9.8)	52.56	10.04	<0.001 ***	
Smoking									0.80
Smoking status									
Current (%)	112,170	77,665 (69.2)	15,763 (14.1)	13,459 (12.0)	5283 (4.7)	2	12		
Case	1175	1014 (86.3)	46 (3.9)	69 (5.9)	46 (3.9)	1	10		
Non-case	110,995	76,651 (69.1)	15,717 (14.2)	13,390 (12.1)	5237 (4.7)	2	12	<0.001 ***	
Former (%)	164,978	111,832 (67.8)	21,185 (12.8)	21,247 (12.9)	10,714 (6.5)	3	24		
Case	1346	1124 (83.5)	59 (4.4)	99 (7.4)	64 (4.8)	3	25		
Non-case	163,632	110,708 (67.7)	21,126 (12.9)	21,148 (12.9)	10,650 (6.5)	3	24	<0.001 ***	
Never (%)	272,310	191,274 (70.2)	33,955 (12.5)	31,269 (11.5)	15,812 (5.8)	4	25		
Case	643	511 (79.5)	46 (7.2)	51 (7.9)	35 (5.4)	4	27		
Non-case	271,667	190,763 (70.2)	33,909 (12.5)	31,218 (11.5)	15,777 (5.8)	4	25	<0.001 ***	
Smoking pack-years [mean (SD)]									
Current <20 years	53,612	35,014 (63.3)	8738 (16.3)	7252 (13.5)	2608 (4.9)	2	12		
Case	278	234 (84.2)	16 (5.8)	19 (6.8)	9 (3.2)	2	16		
Non-case	53,334	34,780 (65.2)	8722 (16.4)	7233 (13.6)	2599 (4.9)	2	12	<0.001 ***	
Current >20 years	47,756	34,730 (72.7)	5665 (11.9)	4969 (10.4)	2392 (5.0)	2	14		
Case	769	665 (86.5)	25 (3.3)	44 (5.7)	35 (4.6)	4	8.2		
Non-case	46,987	34,065 (72.5)	5640 (12.0)	4925 (10.5)	2357 (5.0)	2	14	<0.001 *	
Former <20 years	27,536	23,803 (86.4)	1 (0.0)	1287 (4.7)	2445 (8.9)	9	47		
Case	317	287 (90.5)	0 (0.0)	16 (5.0)	14 (4.4)	9	49		
Non-case	27,219	23,516 (86.4)	1 (0.0)	1271 (4.7)	2431 (8.9)	9	47	0.048 *	
Former >20 years	12,028	10,630 (88.4)	0 (0.0)	499 (4.1)	899 (7.5)	7	44		
Case	393	374 (95.2)	0 (0.0)	9 (2.3)	10 (2.5)	2	14		
Non-case	11,635	10,256 (88.1)	0 (0.0)	490 (4.2)	889 (7.6)	7	44	<0.001 ***	
Current	10,802	7921 (73.3)	1360 (12.6)	1238 (11.5)	283 (2.6)	1.0	4.4		
Case	128	115 (89.8)	5 (3.9)	6 (4.7)	2 (1.6)	0.5	2.3		
Non-case	10.674	7.806 (73.1)	1.355 (12.7)	1.232 (11.5)	281 (2.6)	1.0	4.4	<0.001 ***	
Former	125,414	77,399 (61.7)	21,184 (16.9)	19,461 (15.5)	7370 (5.9)	1.9	7.2		
Case	636	463 (72.8)	59 (9.3)	74 (11.6)	40 (6.3)	1.9	5.7		
Non-case	124,779	76,936 (61.7)	21,125 (16.9)	19,387 (15.5)	7330 (5.9)	1.9	7.2	<0.001 ***	
Never	272,309	191,274 (70.2)	33,954 (12.5)	31,269 (11.5)	15,812 (5.8)	0.53	0.91		
Case	643	511 (79.5)	46 (7.2)	51 (7.9)	35 (5.4)	0.39	0.85		
Non-case	271,666	190,763 (70.2)	33,908 (12.5)	31,218 (11.5)	15,777 (5.8)	0.53	0.91	<0.001 ***	
Ethnicity (%)									0.60
Caucasian	545,443	378,209 (69.3)	70,903 (13.1)	65,521 (12.0)	30,810 (5.6)	3	23		
Case	3150	2639 (83.8)	151 (4.8)	217 (6.9)	143 (4.5)	2	22		
Non-case	542,293	375,570 (69.3)	70,752 (13.0)	65,304 (12.0)	30,667 (5.7)	3	23	<0.001 ***	
Non-Caucasian	3735	2329 (62.4)	0 (0.0)	434 (11.6)	972 (26.0)	14	44		
Case	12	8 (66.7)	0 (0.0)	2 (16.7)	2 (16.7)	6	14		
Non-case	3723	2321	0 (0.0)	432	970	14	45	0.70	
Total energy intake [kcal/day; mean (SD)]	2100 (700)	2100 (700)	1900 (600)	2000 (700)	2200 (800)	2100	700		0.57
Case	2200 (700)	2200 (700)	2100 (500)	2100 (700)	2200 (600)	2200	700		
Non-case	2100 (700)	2100 (700)	1900 (600)	2000 (700)	2200 (800)	2100	700	<0.001 ***	
Vegetable intake [g/day; mean (SD)]	190 (130)	190 (130)	190 (130)	180 (130)	160 (120)	190	130		0.65
Case	210 (140)	210 (140)	200 (130)	170 (110)	160 (100)	210	140		
Non-case	190 (130)	190 (130)	190 (130)	180 (130)	160 (120)	190	130	<0.001 ***	
Fruit intake [g/day; mean (SD)]	120 (110)	130 (120)	106 (95)	108 (97)	100 (100)	120	110		0.39
Case	120 (120)	130 (120)	109 (92)	104 (85)	120 (120)	120	120		
Non-case	120 (110)	130 (120)	106 (95)	108 (97)	100 (100)	120	110	0.051	

*p*-value < 0.05 was considered statistically significant. *p*-interaction < 0.05 was considered statistically significant. N = number; SD = standard deviation; kcal = kilocalorie; g = gram. A calculated by Chi square (X^2^) test for categorical variables and *t* test for continuous variables between bladder cancer cases and non-cases. * *p* < 0.05, *** *p* < 0.001.

**Table 2:** Baseline characteristics of the study population for sub-categories of soy consumption.

Characteristics	Total	No Soy Consumption (0 g/Day)	Light Soy Consumption (>0–<1 g/Day)	Moderate Soy Consumption (≥1–<10 g/Day)	High Soy Consumption (≥10 g/Day)	Mean (g/Day)	SD	*p* Value
Soybean oil intake								
N (%)	451,624	395,044 (87.5)	20,040 (4.4)	17,700 (3.9)	18,840 (4.2)	0.25	0.72	
Case (%)	1650	1518 (92.0)	25 (1.5)	49 (3.0)	58 (3.5)	0.18	0.65	
Non-case (%)	449,974	393,526 (87.5)	20,015 (4.4)	17,651 (3.9)	18,782 (4.2)	0.25	0.72	<0.001 ***
Soybean intake								
N (%)	117,916	65,522 (55.6)	17,866 (15.2)	17,054 (14.5)	17,444 (14.8)	0.9	1.1	
Case (%)	1400	1152 (82.3)	105 (7.5)	71 (5.1)	72 (5.1)	0.33	0.79	
Non-case (%)	116,516	64,400 (55.3)	17,761 (15.2)	16,983 (14.6)	17,372 (14.9)	0.9	1.1	<0.001 ***
Soy milk intake								
N (%)	92,581	88,364 (95.4)	1489 (1.6)	2171 (2.3)	557 (0.6)	0.1	0.4	
Case (%)	637	607 (95.3)	10 (1.6)	17 (2.7)	3 (0.5)	0.1	0.4	
Non-case (%)	91,944	87,757 (95.4)	1479 (1.6)	2154 (2.3)	554 (0.6)	0.1	0.4	0.92
Unfermented soy paste intake								
N (%)	67,874	0 (0.0)	27,463 (40.5)	17,804 (26.2)	22,607 (33.3)	1.9	0.86	
Case (%)	135	0 (0.0)	66 (48.9)	40 (29.6)	29 (21.5)	1.7	0.8	
Non-case (%)	67,739	0 (0.0)	27,397 (40.4)	17,764 (26.2)	22,578 (33.3)	1.9	0.86	0.01 *

*p*-value < 0.05 was considered statistically significant. *p*-interaction < 0.05 was considered statistically significant. N = number; SD = standard deviation; kcal = kilocalorie; g = gram. A calculated by Chi square (X^2^) test for categorical variables and *t* test for continuous variables between bladder cancer cases and non-cases. * *p* < 0.05, *** *p* < 0.001.

**Table 3:** Adjusted hazard ratios and 95% confidence intervals of bladder cancer according to soy consumption level.

Study Subgroup	Model Adjustments	No. Cases/Total Participants	No Consumption HR (95% CI) (Never)	Ever Consumption HR (95% CI)	Light Soy Consumption HR (95% CI)	Moderate Soy Consumption HR (95% CI)	High Soy Consumption HR (95% CI)	*p* Trend
**Overall**	Model 1 ^a^	3164/549,458	Ref	0.86 (0.75–0.99)	0.84 (0.68–1.04)	0.92 (0.77–1.10)	0.82 (0.67–1.00)	
	Model 2 ^b^	3164/549,450	Ref	1.00 (0.87–1.15)	1.05 (0.85–1.29)	1.06 (0.88–1.27)	0.91 (0.74–1.11)	
	Model 3 ^c^	3110/523,965	Ref	1.00 (0.86–1.14)	1.04 (0.84–1.29)	1.04 (0.87–1.25)	0.91 (0.75–1.11)	0.57
**Soybean oil**	Model 1 ^a^	1650/451,630	Ref	1.59 (1.09–2.31)	1.13 (0.66–1.93)	1.85 (1.17–2.92)	1.66 (1.11–2.48)	
	Model 2 ^b^	1650/451,624	Ref	1.49 (1.03–2.16)	1.05 (0.62–1.80)	1.69 (1.08–2.67)	1.57 (1.06–2.33)	
	Model 3 ^c^	1607/427,017	Ref	1.46 (0.99–2.14)	1.12 (0.65–1.92)	1.65 (1.03–2.64)	1.50 (1.00–2.26)	0.03
**Soybeans**	Model 1 ^a^	1400/117,916	Ref	0.79 (0.62–1.01)	0.93 (0.70–1.23)	0.70 (0.51–0.96)	0.72 (0.53–0.99)	
	Model 2 ^b^	1400/117,916	Ref	0.87 (0.68–1.11)	1.05 (0.79–1.38)	0.76 (0.56–1.03)	0.77 (0.56–1.05)	
	Model 3 ^c^	1390/116,985	Ref	0.88 (0.69–1.12)	1.05 (0.80–1.39)	0.76 (0.56–1.04)	0.78 (0.57–1.07)	0.04
**Soy milk**	Model 1 ^a^	637/92,581	Ref	0.80 (0.55–1.16)	1.01 (0.54–1.89)	0.76 (0.47–1.89)	0.57 (0.18–1.76)	
	Model 2 ^b^	637/92,573	Ref	1.02 (0.70–1.48)	1.28 (0.68–2.39)	0.98 (0.60–1.60)	0.71 (0.23–2.21)	
	Model 3 ^c^	633/91,743	Ref	1.00 (0.68–1.46)	1.28 (0.68–2.40)	0.93 (0.56–1.54)	0.72 (0.23–2.24)	0.73
**Unfermented soy paste intake**	Model 1 ^a^	135/67,874			Ref	1.15 (0.69–1.91)	1.01 (0.64–1.58)	
	Model 2 ^b^	135/67,874			Ref	1.18 (0.70–1.99)	1.02 (0.65–1.60)	
	Model 3 ^c^	134/67,829			Ref	1.26 (0.75–2.12)	1.04 (0.65–1.66)	0.83

Reference group was never soy consumers, unless otherwise specified. For analyses restricted to soy consumers, light soy consumers were used as the reference group. HR, hazard ratio, CI, confidence interval. ^a^ Model 1: crude model. ^b^ Model 2: adjusted for age, sex, smoking and energy intake. ^c^ Model 3: additionally adjusted for vegetable and fruit intake. *p* trend < 0.05 was considered statistically significant.

**Table 4:** Adjusted hazard ratios and 95% confidence intervals of bladder cancer according to soy consumption level stratified by sex.

Study Subgroup	Model Adjustments	No. Cases/Total Participants	No Consumption HR (95% CI) (Never)	Ever Consumption HR (95% CI)	Light Soy Consumption HR (95% CI)	Moderate Soy Consumption HR (95% CI)	High Soy Consumption HR (95% CI)	*p* Trend
**Male**								
Overall	Model 1 ^a^	2376/178,424	Ref	0.89 (0.75–1.05)	0.85 (0.64–1.13)	0.93 (0.75–1.15)	0.87 (0.70–1.09)	
	Model 2 ^b^	2376/178,417	Ref	0.98 (0.82–1.16)	0.94 (0.71–1.25)	1.03 (0.83–1.28)	0.95 (0.75–1.19)	
	Model 3 ^c^	2336/174,672	Ref	0.96 (0.81–1.14)	0.93 (0.69–1.23)	1.00 (0.80–1.25)	0.94 (0.75–1.18)	0.67
Soybean oil	Model 1 ^a^	1154/ 132,659	Ref	1.62 (1.00–2.64)	1.29 (0.57–2.95)	1.60 (0.83–3.09)	1.68 (1.01–2.78)	
	Model 2 ^b^	1154/132,659	Ref	1.63 (1.00–2.66)	1.25 (0.54–2.86)	1.61 (0.83–2.80)	1.69 (1.02–2.80)	
	Model 3 ^c^	1123/129,489	Ref	1.52 (0.92–2.51)	1.28 (0.55–2.98)	1.59 (0.81–3.14)	1.53 (0.90–2.58)	0.11
Soybeans	Model 1 ^a^	1129/ 55,678	Ref	0.84 (0.65–1.09)	0.91 (0.66–1.26)	0.79 (0.56–1.11)	0.81 (0.57–1.14)	
	Model 2 ^b^	1129/ 55,678	Ref	0.92 (0.71–1.20)	1.01 (0.74–1.39)	0.87 (0.62–1.22)	0.88 (0.62–1.24)	
	Model 3 ^c^	1120/55,064	Ref	0.93 (0.72–1.22)	1.02 (0.74–1.40)	0.87 (0.62–1.23)	0.89 (0.63–1.26)	0.38
Soy milk	Model 1 ^a^	488/42,897	Ref	0.71 (0.43–1.18)	1.08 (0.48–2.42)	0.65 (0.34–1.28)	0.32 (0.04–2.25)	
	Model 2 ^b^	488/42,890	Ref	0.82 (0.50–1.36)	1.18 (0.53–2.64)	0.78 (0.40–1.52)	0.35 (0.05–2.49)	
	Model 3 ^c^	486/42,353	Ref	0.82 (0.49–1.36)	1.16 (0.52–2.61)	0.78 (0.40–1.52)	0.35 (0.05–2.49)	0.27
Unfermented soy paste intake	Model 1 ^a^	79/18,208			Ref	1.35 (0.68–2.68)	1.15 (0.65–2.05)	
	Model 2 ^b^	79/18,208			Ref	1.42 (0.71–2.84)	1.25 (0.70–2.23)	
	Model 3 ^c^	79/18,186			Ref	1.52 (0.75–3.07)	1.41 (0.77–2.57)	0.24
**Female**								
Overall	Model 1 ^a^	788/371,028	Ref	1.03 (0.80–1.33)	1.17 (0.84–1.64)	1.14 (0.82–1.58)	0.80 (0.54–1.18)	
	Model 2 ^b^	788/371,027	Ref	1.05 (0.82–1.35)	1.18 (0.84–1.65)	1.14 (0.82–1.57)	0.83 (0.56–1.24)	
	Model 3 ^c^	774/349,293	Ref	1.08 (0.83–1.40)	1.21 (0.86–1.71)	1.17 (0.84–1.63)	0.85 (0.57–1.27)	0.87
Soybean oil	Model 1 ^a^	496/318,965	Ref	1.31 (0.76–2.25)	0.93 (0.47–1.84)	1.71 (0.92–3.17)	1.41 (0.76–2.62)	
	Model 2 ^b^	496/318,965	Ref	1.29 (0.75–2.23)	0.92 (0.46–1.84)	1.66 (0.89–3.10)	1.38 (0.74–2.59)	
	Model 3 ^c^	484/297,528	Ref	1.35 (0.76–2.39)	1.00 (0.49–2.04)	1.63 (0.85–3.14)	1.47 (0.77–2.79)	0.14
Soybeans	Model 1 ^a^	271/62,238	Ref	0.67 (0.37–1.21)	1.02 (0.55–1.90)	0.46 (0.22–0.97)	0.45 (0.21–0.96)	
	Model 2 ^b^	271/62,238	Ref	0.68 (0.38–1.22)	0.99 (0.53–1.84)	0.46 (0.22–0.98)	0.48 (0.22–1.02)	
	Model 3 ^c^	270/61,921	Ref	0.69 (0.38–1.24)	1.00 (0.54–1.86)	0.47 (0.22–1.00)	0.48 (0.22–1.04)	0.01
Soy milk	Model 1 ^a^	149/49,684	Ref	1.39 (0.79–2.44)	1.45 (0.54–3.93)	1.34 (0.65–2.80)	1.42 (0.35–5.75)	
	Model 2 ^b^	149/49,683	Ref	1.43 (0.81–2.52)	1.48 (0.54–4.0)	1.39 (0.67–2.90)	1.49 (0.37–6.06)	
	Model 3 ^c^	147/49,390	Ref	1.40 (0.78–2.53)	1.53 (0.56–4.2)	1.30 (0.59–2.83)	1.59 (0.39–6.46)	0.29
Unfermented soy paste intake	Model 1 ^a^	56/49,666			Ref	0.97 (0.44–2.13)	0.81 (0.39–1.66)	
	Model 2 ^b^	56/49,666			Ref	0.98 (0.45–2.15)	0.80 (0.39–1.64)	
	Model 3 ^c^	55/49,643			Ref	1.05 (0.48–2.31)	0.73 (0.34–1.56)	0.44

Reference group was never soy consumers, unless otherwise specified. For analyses restricted to soy consumers, light soy consumers were used as the reference group. HR, hazard ratio, CI, confidence interval. ^a^ Model 1: crude model. ^b^ Model 2: adjusted for age, sex, smoking and energy intake. ^c^ Model 3: additionally adjusted for vegetable and fruit intake. *p* trend < 0.05 was considered statistically significant.

## Data Availability

The data presented in this study are available on request from the corresponding author due to ethical restriction.

## References

[1] WHO Cancer Today. https://gco.iarc.fr/today/home.

[2] Antoni S., Ferlay J., Soerjomataram I., Znaor A., Jemal A., Bray F. (2017). Bladder Cancer Incidence and Mortality: A Global Overview and Recent Trends. Eur. Urol..

[3] Burger M., Catto J.W., Dalbagni G., Grossman H.B., Herr H., Karakiewicz P., Kassouf W., Kiemeney L.A., La Vecchia C., Shariat S. (2013). Epidemiology and risk factors of urothelial bladder cancer. Eur. Urol..

[4] Wada K., Tsuji M., Tamura T., Konishi K., Goto Y., Mizuta F., Koda S., Uji T., Hori A., Tanabashi S. (2018). Soy Isoflavone Intake and Bladder Cancer Risk in Japan: From the Takayama Study. Cancer Epidemiol. Biomark. Prev..

[5] Botteman M.F., Pashos C.L., Redaelli A., Laskin B., Hauser R. (2003). The health economics of bladder cancer. Pharmacoeconomics.

[6] Hong Y.M., Loughlin K.R. (2008). Economic impact of tumor markers in bladder cancer surveillance. Urology.

[7] Busby J.E., Kamat A.M. (2006). Chemoprevention for bladder cancer. J. Urol..

[8] Stenzl A., Hennenlotter J., Schilling D. (2008). Can we still afford bladder cancer?. Curr. Opin. Urol..

[9] De Stefani E., Boffetta P., Ronco A.L., Deneo-Pellegrini H., Acosta G., Mendilaharsu M. (2008). Dietary patterns and risk of bladder cancer: A factor analysis in Uruguay. Cancer Causes Control.

[10] Zeegers M.P., Kellen E., Buntinx F., van den Brandt P.A. (2004). The association between smoking, beverage consumption, diet and bladder cancer: A systematic literature review. World J. Urol..

[11] Letašiová S., Medve’ová A., Šovčíková A., Dušinská M., Volkovová K., Mosoiu C., Bartonová A. (2012). Bladder cancer, a review of the environmental risk factors. Environ. Health.

[12] Leppert J.T., Shvarts O., Kawaoka K., Lieberman R., Belldegrun A.S., Pantuck A.J. (2006). Prevention of bladder cancer: A review. Eur. Urol..

[13] Strope S.A., Montie J.E. (2008). The causal role of cigarette smoking in bladder cancer initiation and progression, and the role of urologists in smoking cessation. J. Urol..

[14] Cumberbatch M.G., Rota M., Catto J.W., La Vecchia C. (2016). The Role of Tobacco Smoke in Bladder and Kidney Carcinogenesis: A Comparison of Exposures and Meta-analysis of Incidence and Mortality Risks. Eur. Urol..

[15] Rink M., Crivelli J.J., Shariat S.F., Chun F.K., Messing E.M., Soloway M.S. (2015). Smoking and Bladder Cancer: A Systematic Review of Risk and Outcomes. Eur. Urol. Focus.

[16] Murta-Nascimento C., Schmitz-Dräger B.J., Zeegers M.P., Steineck G., Kogevinas M., Real F.X., Malats N. (2007). Epidemiology of urinary bladder cancer: From tumor development to patient’s death. World J. Urol..

[17] Piyathilake C. (2016). Dietary factors associated with bladder cancer. Investig. Clin. Urol..

[18] Sarkar F.H., Li Y. (2002). Mechanisms of cancer chemoprevention by soy isoflavone genistein. Cancer Metastasis Rev..

[19] Villanueva C.M., Cantor K.P., King W.D., Jaakkola J.J., Cordier S., Lynch C.F., Porru S., Kogevinas M. (2006). Total and specific fluid consumption as determinants of bladder cancer risk. Int. J. Cancer.

[20] Yu E.Y., Wesselius A., van Osch F., Stern M.C., Jiang X., Kellen E., Lu C.M., Pohlabeln H., Steineck G., Marshall J. (2019). The association between coffee consumption and bladder cancer in the bladder cancer epidemiology and nutritional determinants (BLEND) international pooled study. Cancer Causes Control.

[21] Pelucchi C., La Vecchia C. (2009). Alcohol, coffee, and bladder cancer risk: A review of epidemiological studies. Eur. J. Cancer Prev..

[22] Witlox W.J.A., van Osch F.H.M., Brinkman M., Jochems S., Goossens M.E., Weiderpass E., White E., van den Brandt P.A., Giles G.G., Milne R.L. (2020). An inverse association between the Mediterranean diet and bladder cancer risk: A pooled analysis of 13 cohort studies. Eur. J. Nutr..

[23] Büchner F.L., Bueno-de-Mesquita H.B., Ros M.M., Kampman E., Egevad L., Overvad K., Tjønneland A., Roswall N., Clavel-Chapelon F., Boutron-Ruault M.C. (2011). Variety in vegetable and fruit consumption and risk of bladder cancer in the European Prospective Investigation into Cancer and Nutrition. Int. J. Cancer.

[24] WCRF Bladder Cancer. https://www.wcrf.org/dietandcancer/bladder-cancer.

[25] Chen Z., Zheng W., Custer L.J., Dai Q., Shu X.O., Jin F., Franke A.A. (1999). Usual dietary consumption of soy foods and its correlation with the excretion rate of isoflavonoids in overnight urine samples among Chinese women in Shanghai. Nutr. Cancer.

[26] Zhou J.R., Mukherjee P., Gugger E.T., Tanaka T., Blackburn G.L., Clinton S.K. (1998). Inhibition of murine bladder tumorigenesis by soy isoflavones via alterations in the cell cycle, apoptosis, and angiogenesis. Cancer Res..

[27] Su S.J., Yeh T.M., Lei H.Y., Chow N.H. (2000). The potential of soybean foods as a chemoprevention approach for human urinary tract cancer. Clin. Cancer Res..

[28] Kostelac D., Rechkemmer G., Briviba K. (2003). Phytoestrogens modulate binding response of estrogen receptors α and β to the estrogen response element. J. Agric. Food Chem..

[29] Hsu I., Vitkus S., Da J., Yeh S. (2013). Role of oestrogen receptors in bladder cancer development. Nat. Rev. Urol..

[30] Leonard L.M., Choi M.S., Cross T.L. (2022). Maximizing the Estrogenic Potential of Soy Isoflavones through the Gut Microbiome: Implication for Cardiometabolic Health in Postmenopausal Women. Nutrients.

[31] Singh A.V., Franke A.A., Blackburn G.L., Zhou J.R. (2006). Soy phytochemicals prevent orthotopic growth and metastasis of bladder cancer in mice by alterations of cancer cell proliferation and apoptosis and tumor angiogenesis. Cancer Res..

[32] Vantyghem S.A., Wilson S.M., Postenka C.O., Al-Katib W., Tuck A.B., Chambers A.F. (2005). Dietary genistein reduces metastasis in a postsurgical orthotopic breast cancer model. Cancer Res..

[33] Zhou J.R., Yu L., Zhong Y., Nassr R.L., Franke A.A., Gaston S.M., Blackburn G.L. (2002). Inhibition of orthotopic growth and metastasis of androgen-sensitive human prostate tumors in mice by bioactive soybean components. Prostate.

[34] Pavese J.M., Farmer R.L., Bergan R.C. (2010). Inhibition of cancer cell invasion and metastasis by genistein. Cancer Metastasis Rev..

[35] Sun C.L., Yuan J.M., Wang X.L., Gao Y.T., Ross R.K., Yu M.C. (2004). Dietary soy and increased risk of bladder cancer: A prospective cohort study of men in Shanghai, China. Int. J. Cancer.

[36] Goossens M.E., Isa F., Brinkman M., Mak D., Reulen R., Wesselius A., Benhamou S., Bosetti C., Bueno-de-Mesquita B., Carta A. (2016). International pooled study on diet and bladder cancer: The bladder cancer, epidemiology and nutritional determinants (BLEND) study: Design and baseline characteristics. Arch. Public Health.

[37] Poortvliet E.J., Klensin J.C., Kohlmeier L. (1992). Rationale document for the Eurocode 2 food coding system (version 91/2). Eur. J. Clin. Nutr..

[38] FoodData Central. https://fdc.nal.usda.gov/.

[39] Sun C.L., Yuan J.M., Arakawa K., Low S.H., Lee H.P., Yu M.C. (2002). Dietary soy and increased risk of bladder cancer: The Singapore Chinese Health Study. Cancer Epidemiol. Biomark. Prev..

[40] Mulligan A., Welch A., McTaggart A., Bhaniani A., Bingham S. (2007). Intakes and sources of soya foods and isoflavones in a UK population cohort study (EPIC-Norfolk). Eur. J. Clin. Nutr..

[41] Messina M., Nagata C., Wu A.H. (2006). Estimated Asian adult soy protein and isoflavone intakes. Nutr. Cancer.

[42] Nakamura Y., Tsuji S., Tonogai Y. (2000). Determination of the levels of isoflavonoids in soybeans and soy-derived foods and estimation of isoflavonoids in the Japanese daily intake. J. AOAC Int..

[43] Zock P.L., Katan M.B. (1998). Linoleic acid intake and cancer risk: A review and meta-analysis. Am. J. Clin. Nutr..

[44] Jiang Z.M., Wilmore D.W., Wang X.R., Wei J.M., Zhang Z.T., Gu Z.Y., Wang S., Han S.M., Jiang H., Yu K. (2010). Randomized clinical trial of intravenous soybean oil alone versus soybean oil plus fish oil emulsion after gastrointestinal cancer surgery. Br. J. Surg..

[45] Banni S., Angioni E., Casu V., Melis M.P., Carta G., Corongiu F.P., Thompson H., Ip C. (1999). Decrease in linoleic acid metabolites as a potential mechanism in cancer risk reduction by conjugated linoleic acid. Carcinogenesis.

[46] Adlercreutz H. (2002). Phyto-oestrogens and cancer. Lancet Oncol..

[47] Mahmoud A.M., Yang W., Bosland M.C. (2014). Soy isoflavones and prostate cancer: A review of molecular mechanisms. J. Steroid Biochem. Mol. Biol..

[48] Mirvish S.S. (1995). Role of N-nitroso compounds (NOC) and N-nitrosation in etiology of gastric, esophageal, nasopharyngeal and bladder cancer and contribution to cancer of known exposures to NOC. Cancer Lett..

[49] Loh Y.H., Jakszyn P., Luben R.N., Mulligan A.A., Mitrou P.N., Khaw K.T. (2011). N-Nitroso compounds and cancer incidence: The European Prospective Investigation into Cancer and Nutrition (EPIC)-Norfolk Study. Am. J. Clin. Nutr..

[50] Nagata C., Takatsuka N., Kawakami N., Shimizu H. (2002). A prospective cohort study of soy product intake and stomach cancer death. Br. J. Cancer.

[51] Ko K.P., Park S.K., Yang J.J., Ma S.H., Gwack J., Shin A., Kim Y., Kang D., Chang S.H., Shin H.R. (2013). Intake of soy products and other foods and gastric cancer risk: A prospective study. J. Epidemiol..

[52] Jang H.H., Lee Y.M., Choe J.S., Kwon O. (2021). Validation of soy isoflavone intake and its health effects: A review of the development of exposure biomarkers. Nutr. Res. Pract..

[53] Madeb R., Messing E.M. (2004). Gender, racial and age differences in bladder cancer incidence and mortality. Urol. Oncol..

[54] Fajkovic H., Halpern J.A., Cha E.K., Bahadori A., Chromecki T.F., Karakiewicz P.I., Breinl E., Merseburger A.S., Shariat S.F. (2011). Impact of gender on bladder cancer incidence, staging, and prognosis. World J. Urol..

[55] Koebnick C., Michaud D., Moore S.C., Park Y., Hollenbeck A., Ballard-Barbash R., Schatzkin A., Leitzmann M.F. (2008). Body mass index, physical activity, and bladder cancer in a large prospective study. Cancer Epidemiol. Biomark. Prev..

[56] Clarke R., Shipley M., Lewington S., Youngman L., Collins R., Marmot M., Peto R. (1999). Underestimation of risk associations due to regression dilution in long-term follow-up of prospective studies. Am. J. Epidemiol..

